# Identification and Prediction of Diabetic Sensorimotor Polyneuropathy Using Individual and Simple Combinations of Nerve Conduction Study Parameters

**DOI:** 10.1371/journal.pone.0058783

**Published:** 2013-03-22

**Authors:** Alanna Weisman, Vera Bril, Mylan Ngo, Leif E. Lovblom, Elise M. Halpern, Andrej Orszag, Bruce A. Perkins

**Affiliations:** 1 Division of Endocrinology and Metabolism, Department of Medicine, University of Toronto, Toronto, Ontario, Canada; 2 Division of Neurology, Department of Medicine, University of Toronto, Toronto, Ontario, Canada; Medical University Innsbruck, Austria

## Abstract

**Objective:**

Evaluation of diabetic sensorimotor polyneuropathy (DSP) is hindered by the need for complex nerve conduction study (NCS) protocols and lack of predictive biomarkers. We aimed to determine the performance of single and simple combinations of NCS parameters for identification and future prediction of DSP.

**Materials and Methods:**

406 participants (61 with type 1 diabetes and 345 with type 2 diabetes) with a broad spectrum of neuropathy, from none to severe, underwent NCS to determine presence or absence of DSP for cross-sectional (concurrent validity) analysis. The 109 participants without baseline DSP were re-evaluated for its future onset (predictive validity). Performance of NCS parameters was compared by area under the receiver operating characteristic curve (AROC).

**Results:**

At baseline there were 246 (60%) Prevalent Cases. After 3.9 years mean follow-up, 25 (23%) of the 109 Prevalent Controls that were followed became Incident DSP Cases. Threshold values for peroneal conduction velocity and sural amplitude potential best identified Prevalent Cases (AROC 0.90 and 0.83, sensitivity 80 and 83%, specificity 89 and 72%, respectively). Baseline tibial F-wave latency, peroneal conduction velocity and the sum of three lower limb nerve conduction velocities (sural, peroneal, and tibial) best predicted 4-year incidence (AROC 0.79, 0.79, and 0.85; sensitivity 79, 70, and 81%; specificity 63, 74 and 77%, respectively).

**Discussion:**

Individual NCS parameters or their simple combinations are valid measures for identification and future prediction of DSP. Further research into the predictive roles of tibial F-wave latencies, peroneal conduction velocity, and sum of conduction velocities as markers of incipient nerve injury is needed to risk-stratify individuals for clinical and research protocols.

## Introduction

Diabetic sensorimotor polyneuropathy (DSP) is the most common form of nerve injury in diabetes, with an estimated prevalence of 50% [1], [2]. It may involve motor, sensory, and autonomic nerves and is characterized by a nerve impairment that is symmetrical and length-dependent [3]. These variable attributes of nerve injury and their manifestations challenge diagnostic strategies for DSP. Notwithstanding, the importance of accurate identification of DSP is emphasized by its prediction of all-cause and disease-specific mortality in patients with diabetes, independent of glycemic control [4], [5], [6], [7]. Clinically relevant late stage complications of DSP can be predicted by a single nerve parameter on nerve conduction studies (NCS), and thus NCS are fundamentally the most widely accepted objective test for the diagnosis of DSP and its sequelae [4], [6] [8], [9], [10].

Consensus definitions for DSP consistently recommend a combination of neuropathic symptoms and signs in addition to specific abnormalities in NCS as criteria for diagnosis [11], [12]. Although NCS form the basis for the diagnosis of DSP, they are complex, time-consuming, and require referral to specialized testing centers. In addition, though abnormalities in NCS have been shown to predict foot ulceration, amputation, and mortality, they have not specifically been evaluated for prediction of incipient DSP at a stage that precedes its complications [8], [9], [10].

Despite the limitations in the applicability of NCS in clinical practice, they are the most sensitive, specific, and validated diagnostic test compared to other qualitative and quantitative measures [13], [14], [15], [16], [17], [18], [19]. However, NCS have not been maximally utilized in clinical practice settings or research protocols. Use of simple components of NCS and the analysis of normal and abnormal threshold values specific for patients with diabetes could improve its applicability.

The aim of the current study was to evaluate individual and simple combinations of NCS parameters for cross-sectional performance (concurrent validity) in a cohort of participants with diabetes. Furthermore, for the first time to our knowledge, the role of NCS in prediction of future onset of DSP (predictive validity) – in comparison to the prediction of late-stage complications such as ulceration, amputation and death [4], [6] – was evaluated longitudinally in the subset of participants without DSP at baseline.

## Materials and Methods

### Ethics Statement

The protocol and consent procedures were approved by the Multidisciplinary Research Ethics Board of the Toronto General Hospital Research Institute. All participants provided written informed consent.

478 participants were examined as part of the Toronto Diabetic Neuropathy Cohort between 1999 and 2001 with a baseline assessment [20], [21]. 72 healthy participants without diabetes were excluded from the current analysis leaving a total of 406 participants with diabetes, 345 of which had a diagnosis of type 2 diabetes and 61 of which had a diagnosis of type 1 diabetes. 273 were assessed at follow-up (67%) between 2001 and 2007 with participants having one or two repeat assessments.

### Clinical Stratification Method

Stratified accrual methods that made use of the Toronto Clinical Neuropathy Score (TCNS) have been described previously [20], [21], [22]. In brief, this clinical stratification method was used to ensure a broad spectrum of patients in the study but was not used to define the outcome of DSP. Subjects were graded according to neuropathy severity using 6 symptom scores (the presence or absence of foot pain, numbness, tingling, weakness, imbalance, and upper limb symptoms), 8 reflex scores (bilateral knee and ankle reflexes, each graded as absent, reduced, or normal), and 5 physical examination scores (the presence or absence of pinprick, temperature, light touch, vibration, and position sense) for a total of 19 possible points. Grading was stratified such that ≤5 indicated no neuropathy, 6–8 indicated mild neuropathy, 9–11 indicated moderate neuropathy, and ≥12 indicated severe neuropathy. Accrual into the study was continued until the smallest stratum contained 50 subjects.

### Definition of Prevalent and Incident Cases of DSP

A definition of DSP was developed in accordance with the standard published consensus guidelines for diagnosing DSP [11]. DSP was defined by the presence of at least one neuropathic symptom or sign in addition to electrophysiologic abnormalities in both one sural nerve parameter and one peroneal nerve parameter. Six neuropathic symptoms (pain, numbness, tingling, weakness, ataxia, and upper limb symptoms) and seven neuropathic signs (ankle reflexes, knee reflexes, position sense, and sensation to pinprick, light touch, temperature and vibration) were examined at each visit. Standard reference thresholds were derived from the distribution in healthy populations and the reference values established by the Toronto General Hospital electrophysiology unit, where values less than the 1^st^ or greater than the 99^th^ percentile generally defined abnormality [23].

### Definitions of Electrophysiologic Parameters

NCS were conducted in the electrophysiology lab at Toronto General Hospital using the Counterpoint instrument (Medtronic, Mississauga, Canada) according to the standards of the American Association for Neuromuscular and Electrodiagnostic Medicine and the Canadian Society of Clinical Neurophysiology [24], [25]. Recordings were performed with temperature control (32–34°C), careful distance measurements, and recording of well-defined and artifact-free responses. Latency and amplitude values were read from the equipment after accurate cursor placement was ensured. Distance values were entered into the Counterpoint device and conduction velocities were calculated automatically.

The nerve parameters recorded were: sural sensory nerve action potential amplitude and conduction velocity, peroneal compound muscle action potential amplitude, F-wave latency and conduction velocity, and tibial compound muscle action potential amplitude, F-wave latency and conduction velocity [15], [23]. NCS were typically performed bilaterally with mean values used in statistical analyses. In individuals with only unilateral measurements (for example, in participants with a limb amputation) then the single unilateral value was used. The sums of selected parameters were also examined according to conventional outcome measures used in clinical trials [26]. Sural and tibial amplitude potentials were added for a sum of amplitude potentials. As the unit of measurement for sural amplitude potential (in microvolts) differs from that of tibial and peroneal amplitude potential (in millivolts), some of the summative measures were reported as arbitrary units. Sural, peroneal and tibial conduction velocities were added for a sum of conduction velocities; and peroneal and tibial F-wave latencies were added for a sum of F-wave latencies.

### Clinical and Biochemical Variables

A comprehensive evaluation was performed to exclude other etiologies for neuropathy such as familial, alcoholic, nutritional, and uremic polyneuropathy. This included a general medical exam as well as assessment of neuropathy-related symptoms and signs. Participants completed a questionnaire on clinical factors and comorbidities. Biochemical testing included serum creatinine, complete blood count, glycated hemoglobin A1c, serum lipids, urinary albumin excretion, vitamin B12 and folate levels, serum and urine protein electrophoresis, and thyroid hormone levels [15].

### Data Analysis Plan

#### Concurrent validity

First, a concurrent validity analysis was performed wherein participants with DSP at baseline were classified as Prevalent Cases and participants who did not meet DSP criteria at baseline were classified as Prevalent Controls. In this analysis, individual baseline NCS parameters and simple combinations of these parameters (summations) were used as independent variables for their association with Prevalent Cases compared to Prevalent Controls. In this analysis, 246 of 406 (60%) of participants were classified as Prevalent Cases and 160 of 406 (40%) were classified as Prevalent Controls.

#### Predictive validity

Second, a predictive validity analysis was performed wherein only participants who were not classified as Prevalent Cases at baseline were considered. In this second analysis, individual baseline NCS parameters and simple combinations of these parameters were used as independent variables for association with the subsequent new onset of DSP, termed Incident DSP Cases. Participants who did not meet the case definition for DSP at follow-up were classified as Incident DSP Controls. Of the 160 participants classified as Prevalent Controls, 11 died and 40 were lost to follow-up. The remaining 109 (68%) had follow-up assessment and were included in the predictive validity analysis. Mean follow-up time was 3.9±2.4 years with an interquartile range of 2.1 to 7.1 years. Twenty-five Incident DSP Cases (23%) and 84 Incident DSP Controls (77%) were identified.

### Statistical Analysis

Analyses were performed in SAS (version 9.2 for Windows). Differences in baseline characteristics between groups were analyzed using ANOVA for continuous variables and χ^2^ tests for categorical variables. Student’s t-tests and Chi-Square tests were used to analyze differences in continuous and categorical variables, respectively, between Prevalent Cases and Prevalent Controls in the concurrent validity analysis, as well as between Incident DSP Cases and Incident DSP Controls in the predictive validity analysis. To account for the multiple comparisons owing to inclusion of 11 independent hypotheses in Table 1 (11 nerve conduction parameters or their simple combinations were considered for differences in case-control comparison of nerve function), we maintained the family-wise error rate by considering statistical significance for these tests at α-level <0.0045 using the simple Bonferroni correction method of 0.05/11. To obtain the area under the receiver operating characteristics curve (AROC) and optimal threshold for concurrent and predictive diagnosis of DSP, receiver operating characteristics (ROC) curves were generated. Optimal threshold values were calculated according to the distance formula for two points in the plane, 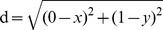 [27], [28]. Comparisons of the AROC for the individual and summative NCS parameters were based on the method of Pencina et al. using two-tailed p values <0.05 [29].

**Table 1:** Baseline Characteristics of the 251 Prevalent DSP Cases and the 107 Prevalent Controls According to the 4-Year Incidence of DSP.

		Prevalent Controls				
	PrevalentCases(n = 246)	IncidentDSPControls(n = 84)	Incident DSP Cases (n = 25)	p value for Prevalent Cases vs. Prevalent Controls	p value for Incident Cases vs. Incident Controls	p value for ANOVA*
**Clinical Characteristics**						
Age (years)	57±10	56±9	55±10	0.04	0.70	0.29
Male Sex (%)	182 (73)	48 (57)	16 (64)	0.001	0.54	0.01
DM duration (years)	14±11	10±11	10±7	0.001	0.98	0.02
Current/Past Smoking (%)	144 (58)	41 (49)	8 (32)	0.0003	0.14	0.02
Alcohol Consumption ≥3 equivalents per day (%)	31 (13)	11 (13)	3 (12)	0.55	0.89	0.98
Type 1 DM (%)	39 (16)	13 (16)	4 (16)	0.77	0.95	0.99
Insulin Use (%)	108 (44)	28 (34)	9 (36)	0.03	0.83	0.21
Oral Hypoglycemic Agent Use (%)	165 (68)	52 (63)	19 (76)	0.92	0.24	0.48
Foot Ulcer History†	19 (8)	2 (2)	0 (0)	0.05	0.44	0.09
Retinopathy History†	58 (24)	8 (9)	3 (12)	0.006	0.72	0.01
Nephropathy History†	44 (18)	11 (13)	8 (32)	0.92	0.03	0.09
TCNS	11.3±3.61	7.95±3.69	8.83±4.09	<0.0001	0.33	<0.0001
**Physical Examination**						
Height (m)	1.72±0.09	1.67±0.09	1.70±0.09	<0.0001	0.19	0.0001
Weight (kg)	88.2±20.9	83.3±16.2	83.4±17.9	0.07	0.98	0.10
BMI (kg/m^2^)	29.8±6.21	29.9±5.15	28.9±5.82	0.70	0.46	0.78
Systolic BP (mmHg)	138.8±17.8	135.6±22.3	130.4±22.3	0.03	0.24	0.05
Diastolic BP (mmHg)	84.4±9.70	83.6±9.89	82.0±12.0	0.25	0.49	0.47
**Laboratory Investigations**						
HbA1c (%)‡	8.4±1.7	7.8±1.7	9.0±1.7	0.16	0.005	0.0074
**Individual NCS Parameters**						
Sural Amp (µV)	2.61±2.23	9.60±5.55	5.74±3.99	<0.0001	0.002	<0.0001
Sural CV (m/s)	39.4±5.51	47.2±5.04	42.2±5.10	<0.0001	<0.0001	<0.0001
Peroneal Amp (mV)	2.60±2.05	6.37±2.58	5.08±2.95	<0.0001	0.04∥	<0.0001
Peroneal CV (m/s)	36.6±5.21	45.0±3.26	41.2±3.60	<0.0001	<0.0001	<0.0001
Peroneal F-wave (ms)	59.5±6.64	49.7±4.46	56.5±9.47	<0.0001	0.003	<0.0001
Tibial Amp (mV)	4.23±3.37	9.34±4.45	7.23±3.83	<0.0001	0.03∥	<0.0001
Tibial CV (m/s)	36.4±5.37	44.8±5.43	40.1±4.17	<0.0001	0.0001	<0.0001
Tibial F-wave (ms)	63.9±7.40	53.6±5.54	59.9±6.08	<0.0001	<0.0001	<0.0001
**Summative NCS Parameters** §						
Amp (arbitrary units)¶	9.49±6.19	25.3±9.80	18.0±7.73	<0.0001	0.001	<0.0001
CV (m/s)	113.8±12.3	137.5±10.2	123.5±9.72	<0.0001	<0.0001	<0.0001
F-wave (ms)	123.2±12.6	103.1±8.99	116.2±14.3	<0.0001	0.0003	<0.0001

Data are means ± standard deviations or *n* (%). For comparisons between two groups, p values reported are χ^2^ test statistics for categorical variables and T-tests for continuous variables. For comparisons between three groups, p values reported are χ^2^ test statistics for categorical variables and ANOVA for continuous variables. Normal values for individual NCS are as follows. Sural amp≥7.2 µV for age ≤65 and ≥5.5 µV for age >65, sural CV≥40 m/s, peroneal amp≥5 µV for age ≤65 and ≥3 for age >65, peroneal CV≥40 m/s, peroneal F wave ≤59 ms for height ≥182.9 cm and ≤58 ms for height ≤182.9 cm, tibial amp≥10 µV, tibial CV≥40 m/s, tibial F wave ≤55 ms.

* p-value for ANOVA between Prevalent Cases, Incident DSP Cases and Incident DSP Controls.

† By subject self-report.

‡ HbA1C, glycated hemoglobin A1C.

§ Summative parameters are composed of the following: sum amplitude = sural+tibial, sum conduction velocity = sural+peroneal +tibial, sum F-wave latency = peroneal+tibial.

¶ Summed amplitude potentials are expressed in arbitrary units since sural amplitude potential is measured in microvolts and tibial amplitude potential is measured in millivolts.

∥ Statistical tests for the NCS parameters applied a Bonferroni correction for multiple comparisons for significance such that p-values <0.0045 (0.05/11) were considered significant. All p-values except for two indicated by this symbol, met significance criteria.

**TCNS, Toronto Clinical Neuropathy Score. Amp, amplitude potential. CV, conduction velocity. F-wave, F-wave latency.**

## Results

The baseline characteristics of the study participants are summarized in Table 1, according to classification as Prevalent Cases, Incident DSP Controls and Incident DSP Cases (Prevalent Controls are comprised of the Incident DSP Controls and Incident DSP Cases). Mean age was similar in all groups, though Prevalent Cases were slightly older. The proportion of males and the duration of diabetes were higher in participants with prevalent DSP. Smoking was more common in Prevalent Cases while there was no difference in alcohol consumption. The proportion of type 1 and type 2 diabetes participants and the use of oral hypoglycemic agents did not differ between groups. Baseline insulin use was higher in Prevalent Cases than Prevalent Controls. Foot ulcer and retinopathy were more common in Prevalent Cases but there was no difference in nephropathy. TCNS was higher in Prevalent Cases than Prevalent Controls, and the mean score is representative of moderate neuropathy in prevalent cases. TCNS was not significantly different between Incident DSP Cases and Incident DSP Controls, though there was a trend toward higher TCNS in Incident DSP Cases. We did not observe differences in BMI between the groups and baseline systolic blood pressure was higher in Prevalent Cases than Prevalent Controls. Though the baseline level of HbA1c was not significantly different between Prevalent Cases and Prevalent Controls (p = 0.16), levels were higher in Incident DSP Cases compared to Incident DSP Controls (p = 0.005). The means of individual and summative NCS parameters within each group are shown in the final sections of Table 1. All 8 individual NCS parameters and summative parameters were different (p<0.0001) between Prevalent Cases and Prevalent Controls at baseline (p-value for Prevalent Cases vs. Prevalent Controls in Table 1). All individual NCS parameters except peroneal and tibial amplitude potentials were different between Incident DSP Controls and Incident DSP Cases (p<0.003, p-value for Incident DSP Controls vs. Incident DSP Cases in Table 1). A test for differences in mean individual and summative NCS parameters between all three groups is also shown (p-value for ANOVA in Table 1).The differences in mean individual NCS parameters between Prevalent Cases, Incident DSP Cases and Incident DSP Controls were further examined graphically to determine the distribution of variables between the three groups. As reflected in the standard deviations of these parameters in Table 1, examination of these plots revealed that substantial overlap in the distributions of individual NCS parameters existed. This overlap of distributions was the least substantial for tibial F-wave latency and tibial conduction velocity.

**Figure 1:**
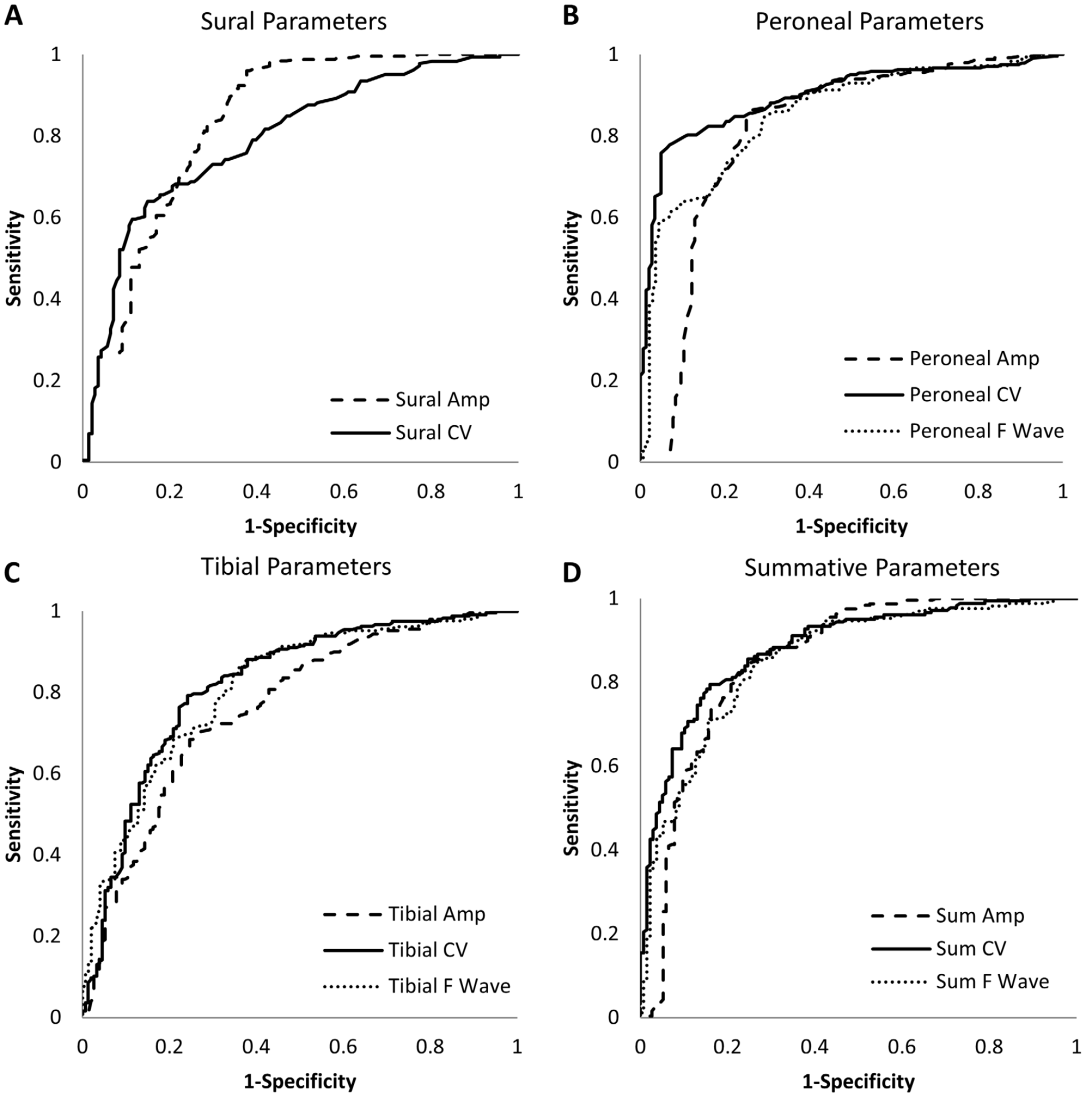
Concurrent validity ROC curves for sural, peroneal, tibial and summative parameters. See Table 2 for estimates of AROC for each parameter. Peroneal conduction velocity and sural amplitude potential had the highest AROC (AROC 0.90 and 0.83, respectively). Dashed lines represent amplitude potentials. Solid lines represent conduction velocities. Dotted lines represent F-wave latencies.

**Figure 2:**
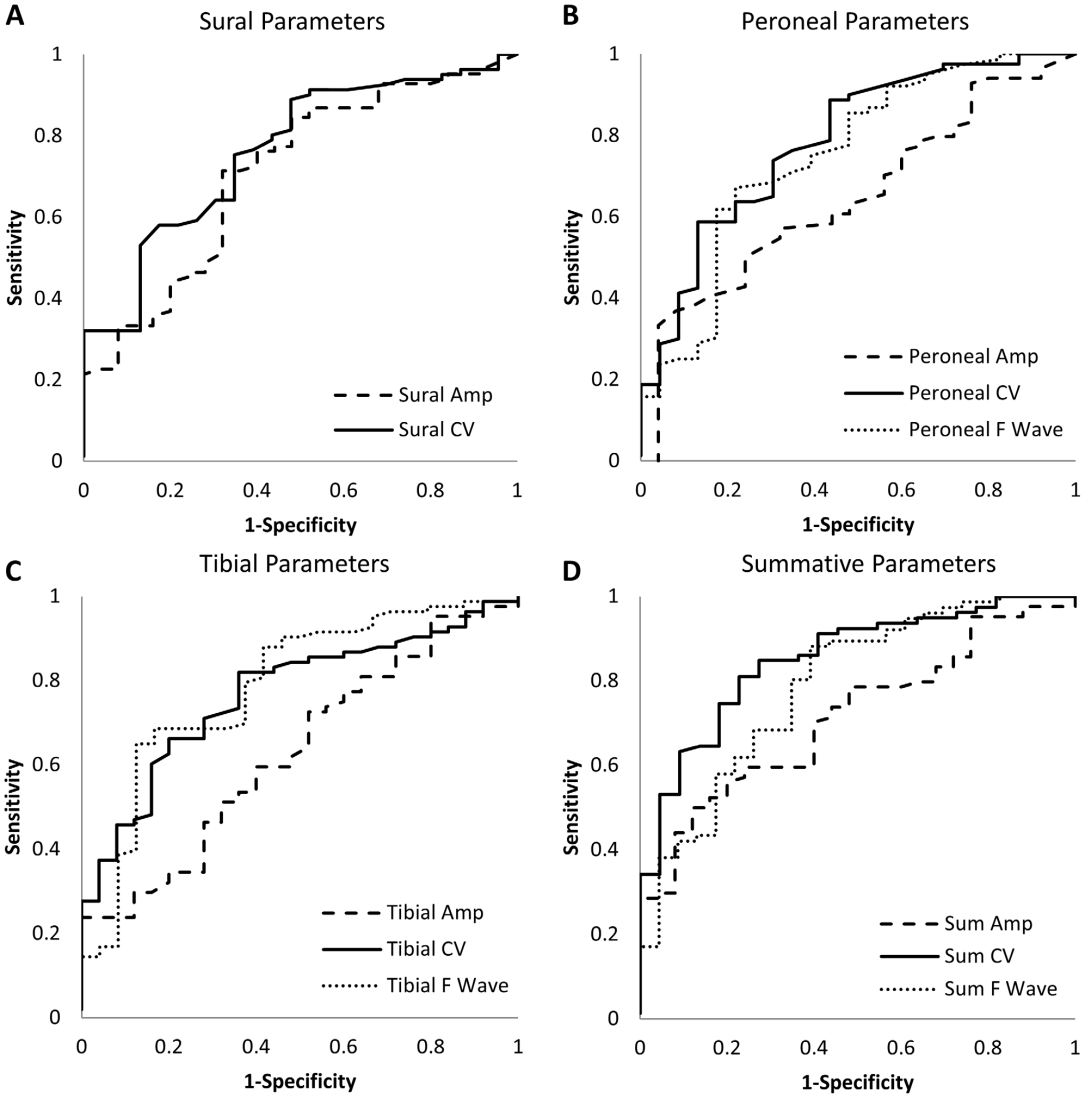
Predictive validity ROC curves for sural, peroneal, tibial and summative parameters. See Table 2 for estimates of AROC for each parameter. Tibial F-wave latency and the sum of sural, peroneal, and tibial conduction velocities had the highest AROC (0.80 and 0.83, respectively). Dashed lines represent amplitude potentials. Solid lines represent conduction velocities. Dotted lines represent F-wave latencies.

To determine if this overlap was sufficiently low to establish a potential diagnostic role, we determined concurrent and predictive diagnostic performance for individual and summative NCS parameters by ROC curve analysis (Figures 1 and 2). The operating characteristics of each test are summarized in Table 2. In the concurrent validity analysis (Figure 1), peroneal conduction velocity had the highest AROC and the ROC curves for the remaining parameters are compared to its curve. All individual NCS parameters had good concurrent diagnostic performance with AROC ranging from 0.76 to 0.90, as summarized in Table 2. Peroneal conduction velocity and sural amplitude potential had the highest operating characteristics, with sensitivities of 80 and 83% and specificities of 89 and 72% at thresholds of 40.4 m/s and 4.9 µV, respectively, for the diagnosis of prevalent DSP. These thresholds were in the low range of normal for these parameters [23]. The operating characteristics of summative parameters were equivalent – but not superior to – peroneal conduction velocity and sural amplitude potential. Thresholds identified by ROC curve analysis corresponded well to standard thresholds except for tibial and sural amplitude potentials and peroneal F wave latency where thresholds identified in the concurrent validity analysis were lower than standard reference thresholds for these parameters.

**Table 2:** Comparison of Area Under the Receiver Operating Characteristic Curve (AROC) Between Individual and Summative NCS Parameters for the Cross-Cectional (Concurrent Validity) Analysis and the Longitudinal (Predictive Validity) Analysis.

					Operating characteristics of the optimal threshold values	
Test	Area under the ROCcurve	*P* Value*	Standard Threshold Values for Normality†	Optimal Threshold Values	Sensitivity	Specificity
**Concurrent Validity**						
Peroneal CV (m/s)	0.90	–	>40	40.4	0.80	0.89
Individual NCS Parameter						
Sural Amp (µV)	0.83	0.36	Age ≤65: >7.2	4.9	0.83	0.72
			Age >65: >5.5			
Sural CV (m/s)	0.80	<0.0001	>40	42.1	0.68	0.79
Peroneal Amp (mv)	0.81	0.002	Age ≤65: >5	4.7	0.84	0.75
			Age >65:>3			
Peroneal F-wave (ms)	0.85	0.0005	Ht≥182.9: <59	53.6	0.85	0.71
			Ht <182.9: <58			
Tibial Amp (mV)	0.76	<0.0001	>10	5.7	0.68	0.75
Tibial CV (m/s)	0.82	<0.0001	>40	40.5	0.79	0.76
Tibial F-wave (ms)	0.81	<0.0001	<58.5	58.5	0.78	0.69
Summative NCS Parameter						
Amp (arbitrary units)	0.86	0.87	Age ≤65: >17.2	16.5	0.83	0.76
			Age <65: >15.5			
CV (m/s)	0.89	0.47	>120	123.3	0.80	0.84
F-wave (ms)	0.86	0.04	Ht≥182.9: <114	112.1	0.84	0.74
			Ht <182.9: <113			
**Predictive Validity**						
Tibial F Wave (ms)	0.79	–	<58.5	57.6	0.79	0.63
Individual NCS Parameter						
Sural Amp (µV)	0.71	0.08	Age ≤65: >7.2	6.8	0.71	0.68
			Age >65: >5.5			
Sural CV (m/s)	0.76	0.32	>40	44.1	0.75	0.65
Peroneal Amp (mV)	0.65	0.0006	Age ≤65: >5	6.2	0.57	0.68
			Age >65: >3			
Peroneal CV (m/s)	0.79	0.89	>40	42.4	0.74	0.70
Peroneal F Wave (ms)	0.75	0.87	Ht≥182.9: <59	51.8	0.67	0.78
			Ht <182.9: <58			
Tibial Amp (mV)	0.63	0.0005	>10	8.4	0.57	0.60
Tibial CV (m/s)	0.77	0.06	>40	41.4	0.80	0.64
Summative NCS Parameter‡						
Sum Amp (arbitrary units)	0.71	0.03	Age ≤65: >17.2	22.8	0.59	0.76
			Age <65: >15.5			
Sum CV (m/s)	0.85	0.15	>120	129.1	0.81	0.77
Sum F Wave (ms)	0.79	0.09	Ht≥182.9: <114	110.1	0.80	0.65
			Ht <182.9: <113			

Normal values for individual NCS are as follows. Sural amp≥7.2 µV for age ≤65 and ≥5.5 µV for age >65, sural CV≥40 m/s, peroneal amp≥5 µV for age ≤65 and ≥3 for age >65, peroneal CV≥40 m/s, peroneal F wave ≤59 ms for height ≥182.9 cm and ≤58 ms for height ≤182.9 cm, tibial amp≥10 µV, tibial CV≥40 m/s, tibial F wave ≤55 ms.

* Two tailed p value for comparison with the AROC for the parameters with the highest AROC in concurrent and predictive analyses.

† Established by the distribution in healthy control subects [23].

‡ Summative parameters are composed of the following: sum amplitude = sural+tibial, sum conduction velocity = sural+peroneal +tibial, sum F-wave latency = peroneal+tibial. Summed amplitude potentials are expressed in arbitrary units since sural amplitude potential is measured in microvolts and tibial amplitude potential is measured in millivolts.

**Amp, amplitude potential. CV, conduction velocity. F-wave, F-wave latency.**

In the predictive validity analysis (Figure 2), tibial F-wave latency and peroneal CV had the highest AROC of the individual NCS parameters and the ROC curves for the remaining parameters are compared to the curve for tibial F-wave latency (Table 2). Tibial and peroneal amplitudes had the lowest AROC of 0.63 and 0.65, respectively. All other individual NCS parameters had good operating characteristics with AROC ranging from 0.71 to 0.77. The individual NCS parameters with the highest AROC were tibial F-wave latency with a sensitivity of 79% and specificity of 63% at a threshold of 57.6 ms, and peroneal conduction velocity with a sensitivity of 74% and specificity of 70% at a threshold of 42.4 m/s. Sum of conduction velocities had a higher sensitivity and specificity (81 and 77%, respectively) than all individual parameters at a threshold of 129.1 m/s. The thresholds identified for prediction of Incident DSP were less abnormal than those thresholds for concurrent diagnosis of DSP and they were still within the normal range by standard thresholds [23].

The parameters with the highest AROC, sensitivities and specificities were further analyzed for their diagnostic operating characteristics. The positive predictive values of peroneal conduction velocity and sural amplitude potential for concurrent DSP were 86 and 81%, respectively, and their negative predictive values were both 73%. The positive predictive values of tibial F-wave latency, peroneal conduction velocity and summative conduction velocity for incident DSP were 47, 42 and 49% and the negative predictive values were 88, 89 and 93%, respectively, using thresholds identified in the predictive validity analysis. The test characteristics of sural amplitude potential and conduction velocity were also analyzed for predictive performance. The positive predictive value of abnormalities in sural amplitude potential and sural conduction velocity for incident DSP were 41 and 43%, respectively, while the negative predictive values were both 88%. A combination of both normal sural amplitude potential and sural conduction velocity (‘negative’ test results) had a negative predictive value of 98%, while the combination of both abnormal sural amplitude potential and sural conduction velocity (‘positive’ test results) had a positive predictive value of 45% for incident DSP.

## Discussion

In a cohort of 406 participants with type 1 and type 2 diabetes, we were able to evaluate the validity of individual and simple combinations of NCS parameters not only for cross-sectional performance in a large diabetes cohort, but also to evaluate their performances in predicting the 4-year onset of future incident DSP. Although individual NCS parameters performed well in the cross-sectional identification of DSP, our primary interest was detection of incident DSP. Thresholds for tibial F-wave latency, peroneal conduction velocity and sum of conduction velocities were identified for 4-year prediction of DSP, with sensitivities approaching 80% and specificities in the range of 70%. This implies simple NCS protocols can reasonably be considered for use in clinical practice and research protocols for the diagnosis of DSP and identification of patients at highest risk for developing incident DSP.

Whereas diabetic retinopathy and nephropathy may be predicted on the basis of micoalbuminuria and fundoscopic examinations, DSP lacks a comparable objective test [30]. Risk factors such as duration of diabetes, glycemic control, hypertension, smoking, obesity and triglycerides have been implicated in DSP incidence; however, these are not diagnostic tests and specific threshold values for prediction have not been identified [8], [9], [10], [31], [32]. In contrast, NCS are objective tests that predict mortality, and peroneal conduction velocity has specifically been shown to predict 6-year risk of foot ulceration and amputation [5], [6], [7]. Our analysis thus fills a void in the paradigm of DSP prediction by identifying an objective test that predicts incipient DSP according to gold-standard methods [11].

In the current study, simple combinations of NCS parameters were superior for detection of incident DSP, but the advantage of these combinations did not appear to apply to the concurrent identification of DSP. We interpret this finding to represent that many individual parameters are sufficient for identifying the more advanced nerve function abnormalities that are present in those individuals with established DSP. However, simple combinations of nerve parameters may enhance the detection of incipient nerve injury which is characterized by more subtle electrophysiological abnormalities. We have previously demonstrated that simple combinations of NCS (sum of lower limb conduction velocities and amplitude potentials) correlate with a clinical scoring system for DSP [20]. Refined NCS combinations used by Dyck and others, in which normal deviates of NCS attributes are added, have higher sensitivity compared to individual parameters [33], [34]. However, in the aforementioned study, associations rather than diagnostic validity were analyzed, and the inclusion of increasing numbers of up to 6 parameters did not further improve sensitivity. This may indicate that for the majority of patients with DSP, once nerve function is unequivocally abnormal, the determination of additional electrophysiological abnormalities does not improve diagnostic performance. Conversely, it implies that in the setting of normal parameters, simple combinations of NCS parameters enhance detection of incipient nerve injury associated with the later incidence of DSP, compared to individual parameters.

We report putative NCS thresholds for overt and incipient DSP. Standard cross-sectional thresholds have been widely reported but are based on percentile distributions in a healthy population, rather than in patients with diabetes [11], [35], [36]. For most individual NCS parameters, the threshold for prediction was slightly less abnormal than the cross-sectional threshold, indicating that these levels represent incipient degrees of injury. For some, such as sural conduction velocity, the threshold for prediction was significantly higher than the standard reference threshold (44.1 versus 40 m/s). Tibial F-wave latency and peroneal conduction velocity were the better-performing individual parameters for prediction. The threshold for peroneal conduction velocity was slightly higher than the standard cross-sectional threshold (42.4 m/s compared to 40 m/s). We see putative advantage to the measurement of peroneal conduction velocity in screening protocols for DSP given our finding that one threshold level performed well in identifying DSP while a slightly higher threshold level for this parameter identified those individuals at highest risk of future DSP. This is supported by a previous study which found peroneal conduction velocity to be the preferred parameter for future prediction of foot ulceration [6]. The threshold for tibial F-wave latency was just slightly lower than the standard cross-sectional threshold (57.6 m/s compared to 58 m/s). F-wave latency is measured following supramaximal stimulation to the distal nerve when an antidromic signal causes a second motor potential. It involves measurement over a longer segment of nerve which may contribute to its higher sensitivity. Lower limb F-wave latencies have previously been shown to be very sensitive for DSP as well as highly reproducible [37], [38]. F-wave latencies demonstrate less variability as a result of temperature, age and height which is particularly important in serial measurements for prediction.

We subsequently analyzed the positive and negative predictive values for the parameters that performed best in ROC curve analysis using our identified thresholds in both the concurrent and predictive validity analyses. Our primary interest was in the predictive validity analysis as this is an area which currently has limited evidence. The results of this analysis demonstrated high negative predictive values but limitations in positive predictive values. As sural parameters are affected earliest in the course of DSP we also analyzed positive and negative predictive values for sural amplitude potential and conduction velocity [39]. When both sural amplitude potential and conduction velocity were normal, the 4-year risk of incident DSP was essentially negligible at 2%. However, the risk when both sural parameters were abnormal was only 45%. These results imply that single or simple combinations of NCS parameters can be used to subdivide patients into those in whom the 4-year risk of incident DSP is exceedingly small and those in whom this risk is nearly 50%, which would enhance risk stratification for clinical practice and enrollment in research studies even if the main performance characteristic is to rule out likelihood of future DSP onset.

There are some limitations to this study. Our amplitude potential and conduction velocity thresholds for prediction of incident DSP were not age- and height-adjusted and thus the precise threshold values that define Incident DSP Cases require further study [33], [40]. Furthermore, we recognize that risk factors such as height may influence NCS independent of their associations with DSP and partially explain their diagnostic accuracy. Height is an independent risk factor for DSP and the performances of F-wave latencies may in part reflect the increased sensitivity of F-wave latencies on account of height. The best individual NCS parameters for cross-sectional diagnosis were sural amplitude potential and peroneal conduction velocity, and this may partially reflect colinearity with the case definition. As the best performing incident DSP parameters did not include the majority of NCS parameters for sural and peroneal nerves, the predictive validity analysis is likely to be least affected by colinearity.

In summary, individual NCS parameters or their simple combinations are sufficiently valid measures for identification and future prediction of DSP. Our findings demonstrate for the first time the ability to identify patients at highest risk for incident DSP by way of alternate threshold values that differ from the normal distributions of NCS parameters. Further research should focus on the specific thresholds for tibial F-wave latency, peroneal conduction velocity and sum of conduction velocities as markers of incipient nerve injury as well as the development of point-of-care NCS tools that could be employed to best identify high-risk individuals for clinical and research protocols.
